# Searching for druggable targets

**DOI:** 10.7554/eLife.107757

**Published:** 2025-07-02

**Authors:** Lana Heganovic, Luiz FM Passalacqua

**Affiliations:** 1 Department of Microbiology and Cell Science, University of Florida Gainesville United States

**Keywords:** shapemap, RNA, siRNA, G-quadruplex, Other

## Abstract

A strategy that analyzes the structural properties of RNA could help identify regions that are promising targets for antiviral drugs.

**Related research article** Luo D, Zheng Y, Huang Z, Wen Z, Guo L, Deng Y, Li Q, Bai Y, Haider S, Wei D. 2025. Exploiting functional regions in the viral RNA genome as druggable entities. *eLife* **13**:RP103923. doi: 10.7554/eLife.103923.

All living organisms and viruses depend on RNA molecules to survive. As well as being the blueprint from which all proteins are made, RNA molecules also regulate protein synthesis, gene expression, and other cellular processes. This makes them an attractive therapeutic target, as compounds that bind to and disrupt the activity of specific RNAs could be used to manipulate currently ‘undruggable’ proteins.

However, creating compounds that target specific RNA molecules is challenging. This is because RNAs are very dynamic: they can adopt several structures, vary greatly in length (ranging from less than 20 to several thousands of nucleotides), and their function and structure are not always conserved across species (Cech and Steitz, 2014; Spitale and Incarnato, 2023). Furthermore, for an RNA-targeting drug to be successful, it needs to interact with regions that are conserved, functional, and physically accessible – not just in vitro, but also in living cells (Childs-Disney et al., 2022). Now, in eLife, Dengguo Wei and co-workers – including Dehua Luo, Yingge Zheng, and Zhiyuan Huang as joint first authors – report a strategy for identifying targetable regions within viral RNA (Luo et al., 2025).

Some viruses use RNA as their genetic material, making their RNA sequences a potential target for antiviral drugs that could prevent replication and transmission. These RNA genomes consist of both coding and non-coding regions, which vary in structure and in how well they are conserved across different viral strains (Payne, 2017). Luo et al. set out to find if a technique called SHAPE-MaP (short for Selective 2′-Hydroxyl Acylation and Primer Extension-Mutational-Profiling) could be used to identify targetable regions in porcine epidemic diarrhea virus (PEDV) – a 28 thousand nucleotide RNA virus that poses a serious threat to pig farming.

SHAPE-MaP provides information on RNA structure using a chemical probe that is more reactive at flexible sites, like RNA loops, and less reactive at more structured regions, such as those that engage in nucleotide base pairing (Siegfried et al., 2014). The resulting data can also be used to calculate a metric called Shannon entropy, which indicates structural stability: the higher the entropy, the lower stability, and the more likely a region is to adopt alternative conformations (Huynen et al., 1997; Siegfried et al., 2014).

The team (who are based at Huazhong Agricultural University and University College London) adapted the SHAPE-MaP method to study the RNA genome of PEDV within infected cells. This approach allowed Luo et al. to classify RNA regions into four groups based on whether they had high or low levels of SHAPE reactivity and Shannon entropy (Figure 1). Further experiments focused on regions with high SHAPE reactivity because those regions tend to be less structured and their nucleotides are more accessible, making them better targets for drugs.

**Figure 1.**
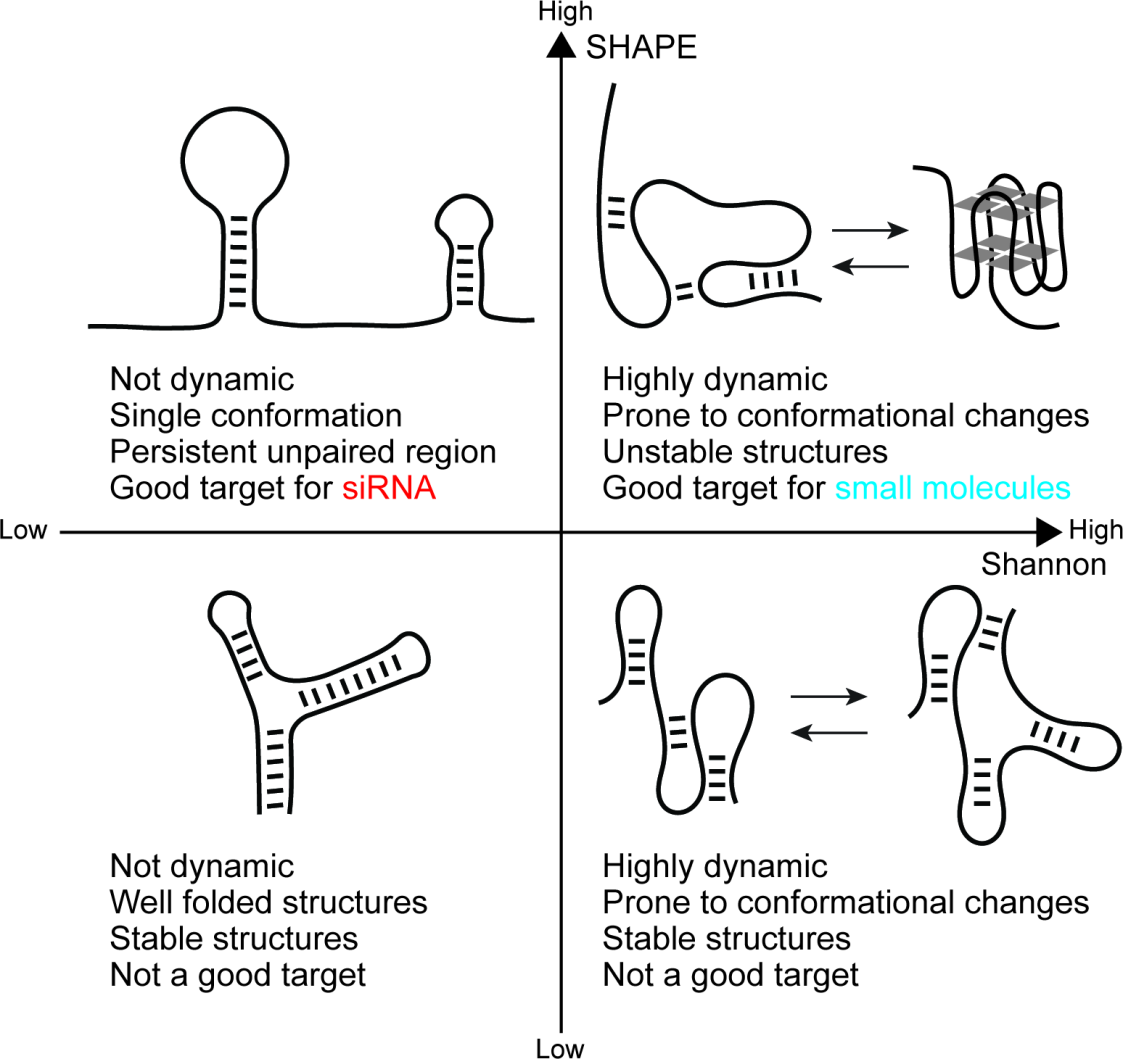
Four categories of RNA sequences in porcine epidemic diarrhea virus. RNA consists of single strands of nucleotides (black line) that can fold into secondary structures, resulting in double-stranded segments (indicated by connecting lines between the single strands). Luo et al. categorized regions of the RNA genome of PEDV into four groups based on their SHAPE reactivity (which reflects nucleotide accessibility and flexibility) and Shannon entropy (which indicates structural stability). (Top left) Regions with high SHAPE reactivity and low Shannon entropy contain stable structures with accessible and flexible nucleotides – like RNA loops and long stretches of unpaired nucleotides – that are good targets for siRNA molecules which can silence genes the virus needs to proliferate. (Top right) Regions with high SHAPE reactivity and high Shannon entropy are flexible and prone to adopting different shapes (as shown by the arrow), such as forming G-quadruplexes (structures containing four strands of RNA that are rich in guanine; right). These regions are promising targets for small molecules that can trigger or stabilize certain conformations that can block RNA replication. (Bottom left) Regions with low SHAPE reactivity and low Shannon entropy are highly folded and stable, making them poor drug targets due to limited accessibility of the RNA. (Bottom right) Regions with low SHAPE reactivity and high Shannon entropy regularly switch shapes, but into stable structures with limited accessibility that are not effective drug targets. PEDV: porcine epidemic diarrhea virus; siRNA: small interfering RNA molecules.

Image Credit: Luiz F. M. Passalacqua.

Regions that display both high SHAPE reactivity and high Shannon entropy are flexible and can adopt alternative conformations that influence the behavior of RNA. Luo et al. were particularly interested in identifying regions that can fold into G-quadruplexes, stable four-stranded structures made up of stacked guanine-rich RNA or DNA sequences (Gellert et al., 1962). These regions are therapeutically attractive as specific molecules can lock them into the G-quadruplex conformation and potentially inhibit processes that involve RNA, such as viral replication (Neidle, 2017). Luo et al. found one G-quadruplex-forming sequence in the RNA genome of PEDV. When cells infected with the virus were treated with Braco19 – a small molecule that promotes G-quadruplex formation – the virus was no longer able to synthesize RNA and proliferation was effectively halted. However, when this sequence was mutated to prevent G-quadruplex formation, Braco-19 no longer caused this inhibitory effect.

Luo et al. then turned their attention to high SHAPE–low entropy regions which are more stable but still accessible, making them potential targets for small interfering RNA (siRNA) molecules that can silence the expression of specific genes (Fire et al., 1998). This revealed four regions with these properties, which were also conserved across multiple PEDV strains. When siRNAs designed to target these sequences were introduced into infected cells, viral proliferation was inhibited. Furthermore, siRNAs that targeted other regions in the PEDV genome did not exhibit this effect.

Taken together, these findings highlight how the SHAPE-MaP technique in combination with Shannon entropy can be used to identify desirable structural features of RNAs in living cells. The work of Luo et al. has also identified new druggable RNA regions within the genome of PEDV, which may also be present in other RNAs of interest.

Recent advances in our understanding of RNA will be crucial for proving that they are indeed feasible therapeutic targets. Strategies like the one presented by Luo et al. – in combination with previous studies and the many more yet to come – will be pivotal in ensuring the success of effective, selective, and safe RNA-targeting drugs. The opportunities are plenty, and the future is hopeful.
